# Interpretive analysis of 85 systematic reviews suggests that narrative syntheses and meta‐analyses are incommensurate in argumentation

**DOI:** 10.1002/jrsm.1231

**Published:** 2016-11-17

**Authors:** G. J. Melendez‐Torres, A. O'Mara‐Eves, J. Thomas, G. Brunton, J. Caird, M. Petticrew

**Affiliations:** ^1^ Warwick Evidence, Division of Health Sciences, Warwick Medical SchoolUniversity of WarwickCoventryUK; ^2^ EPPI‐Centre, Social Science Research Unit, UCL Institute of EducationUniversity College LondonLondonUK; ^3^ Department of Social and Environmental Health ResearchLondon School of Hygiene and Tropical MedicineLondonUK

**Keywords:** systematic review, meta‐analysis, narrative synthesis, interpretive analysis

## Abstract

Using Toulmin's argumentation theory, we analysed the texts of systematic reviews in the area of workplace health promotion to explore differences in the modes of reasoning embedded in reports of narrative synthesis as compared with reports of meta‐analysis. We used framework synthesis, grounded theory and cross‐case analysis methods to analyse 85 systematic reviews addressing intervention effectiveness in workplace health promotion. Two core categories, or ‘modes of reasoning’, emerged to frame the contrast between narrative synthesis and meta‐analysis: practical–configurational reasoning in narrative synthesis (‘what is going on here? What picture emerges?’) and inferential–predictive reasoning in meta‐analysis (‘does it work, and how well? Will it work again?’). Modes of reasoning examined quality and consistency of the included evidence differently. Meta‐analyses clearly distinguished between warrant and claim, whereas narrative syntheses often presented joint warrant–claims. Narrative syntheses and meta‐analyses represent different modes of reasoning. Systematic reviewers are likely to be addressing research questions in different ways with each method. It is important to consider narrative synthesis in its own right as a method and to develop specific quality criteria and understandings of how it is carried out, not merely as a complement to, or second‐best option for, meta‐analysis. © 2016 The Authors. *Research Synthesis Methods* published by John Wiley & Sons Ltd.

## 1 Introduction

When considering how to pool the results of studies included in a systematic review of intervention effectiveness, authors are often faced with a choice of two approaches: meta‐analysis – which is the statistical combination of study findings to estimate a pooled effect – or narrative synthesis, which refers in its most basic form to a descriptive written summary of included studies and their findings (Petticrew and Roberts, 2006). In this work, we primarily focus on narrative synthesis as a method to summarize by using words, rather than statistical methods resulting in a pooled effect, evidence relating to the effectiveness of an intervention. This could include identifying outcome patterns relating to the direction and magnitude of an effect, or tabulating study findings and grouping studies by key study or intervention characteristics (e.g. intervention context or key components) to facilitate understanding. We are less interested in the synthesis ‘method’ known as ‘vote‐counting’, where the number of statistically significant results is counted against the number of statistically non‐significant results, as this method has been shown to be misleading and frequently wrong, through its myopic focus on *p*‐values above or below a cut‐off, usually *p* < 0.05 (Borenstein *et al*., 2009; Hedges and Olkin, 1980).

Opinion is divided about the role of narrative synthesis in systematic reviews. On the one hand, narrative synthesis and meta‐analysis play complementary roles, and depending on user needs, one method may be preferable to the other. When narrative synthesis ‘tells the story’ of the evidence, it can render dense quantitative data intelligible and can increase the policy readiness of a systematic review (Popay *et al*., 2006). Similarly, meta‐analysis can synthesize across multiple narrative study reports to yield a summary effect. On the other hand, some methodological guidance on systematic reviewing, particularly from the perspective of clinical effectiveness, has emphasized narrative synthesis as a second‐best option to meta‐analysis (Achana *et al*., 2014). For example, the Cochrane Handbook notes that ‘when pooling [i.e. meta‐analysis] is judged not to be appropriate’, authors may undertake narrative synthesis, although these ‘are, however, problematic, because it is difficult to set out or describe results without being selective or emphasizing some findings over others’ (Reeves *et al*., 2011). Systematic reviewers may consider a meta‐analysis to be inappropriate for many reasons related to the included studies, such as disparate interventions or dissimilarity of outcome measures and follow‐up times (Petticrew *et al*., 2013; Petticrew *et al*., 2013), thereby opening up numerous opportunities for narrative synthesis to be used as a ‘substitute method’ within conventional guidelines. Others have suggested that a meta‐analysis can be undertaken even in the face of substantial heterogeneity with appropriate attention to dispersion around the mean effect size (Borenstein *et al*., 2009).

### 1.1 Practical arguments: structure and definitions

Although both methods ostensibly aim to present a picture of intervention effectiveness, it remains unclear and unexamined if narrative syntheses answer the same questions by using the same intuitive and cognitive ‘tools’ for reading and synthesizing studies as meta‐analyses do. That is to say, it may not be meaningful to compare one against the other on the same terms. One way to understand the differences between these two methods is to examine the structures of the ‘practical arguments’ advanced in each type of synthesis. As originally suggested by Toulmin (2003), practical arguments are arguments where the goal is to understand the justification of claims advanced in specific situations by using data and observations to test and develop these claims. This is in contrast to arguments that use first principles to develop overarching laws, that is, absolutist reasoning to understand ‘general truths’ about nature. Although Toulmin's work originates from the field of philosophy, it has been popularly used in the fields of communications and rhetoric, where it has long been an influential framework to understand argumentation (Craig, 1999); the Toulmin framework continues to be used today in empirical investigations relating to communication in science and public health (Gray and Kang, 2012; Labrie and Schulz, 2014; Balgopal *et al*., 2016; Whithaus, 2012).

**Figure 1:**
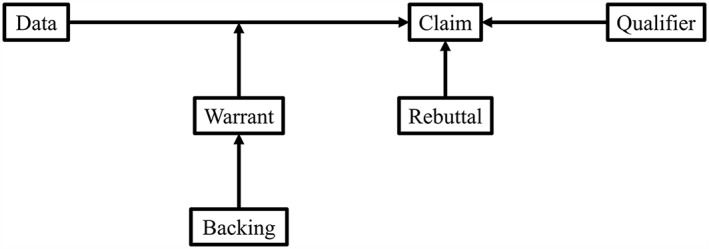
The structure of practical arguments (Toulmin, 2003).

Practical arguments can be understood through their components, which include a ground (or data used), a claim (or a conclusion that is set forth) and a warrant (or the ‘bridge’ that links the ground and the claim). For example, in a primary study of an intervention, outcome data form a ground. A claim that an intervention ‘works’ would need to be supported by a warrant – perhaps a statistical test of effectiveness – linking outcome data to the claim. Additionally, arguments may contain qualifiers, which limit the certainty with which the claim is made (was the effect estimated with precision, or was the evaluation strong or weak?); backing, which is an assertion that supports the warrant itself (were the statistical tests especially robust?); and rebuttal, or statements to restrict the claim (does the intervention generalize only to certain settings?). The structure of practical arguments is summarized in Figure 1 and in Table 1. To develop a hypothetical example, a randomized controlled trial of a smoking cessation intervention would cite a difference in rate of quit attempts as a warrant for a claim that the intervention ‘works’. A qualifier might include that the design was randomized – in support of the warrant – or that confidence intervals were imprecise, to limit certainty of the claim. A backing might note that the statistical tests used appropriately matched the type and distribution of the outcome data, e.g. by using a Poisson distribution to model count of quit attempts instead of treating number of attempts as a continuous variable, and a rebuttal might point out that the intervention was conducted in prisons and thus the findings may not be generalizable to primary care.

**Table 1:** Components of practical arguments (Toulmin, 2003)

Component	Definition
Claim	The conclusion of an argument; a statement requiring support
Ground (often called ‘data’)	Information used to support a claim
Warrant	Proposition used to link the ground and the claim; Toulmin notes that these statements are frequently logical (if A, then B) or implicit.
Backing	Proposition used to support the credibility of the warrant
Qualifier	Proposition that attenuates the certainty with which a claim is made
Rebuttal	Proposition that restricts the conditions under which a claim is applicable

### 1.2 Objective

Viewed through this lens, to ask if narrative syntheses answer the same questions in the same ways as meta‐analyses do is to ask if narrative syntheses make different types of warrant and claim than meta‐analyses do, not – as some methodological guidance for meta‐analysis would suggest – weaker warrants and claims. Our objective in this analysis was to explore differences in the modes of reasoning embedded in reports of narrative synthesis as compared with reports of meta‐analysis. To address this objective, we used the building blocks of Toulmin's argumentation theory to analyse the texts of systematic reviews in the area of workplace health promotion. Throughout our analysis, we view synthesis not only as an end product of an analysis but as a process of reasoning and arguing through different study reports in a systematic review.

## 2 Methods

We used a sample of 106 systematic reviews on workplace health promotion interventions published in English after 1995 that were collected as part of a separate project (Brunton *et al*., 2016). Search and retrieval methods are reported in detail in File S1. We examined a subset of 85 systematic reviews for which electronic full text was available and that specifically synthesized quantitative evaluations of intervention effectiveness.

### 2.1 Methodological rationale

Our approach to coding and synthesis was structured initially by framework synthesis (Ritchie and Spencer, 1994; Oliver *et al*., 2008) and subsequently by grounded theory oriented in a pragmatist epistemology (Corbin and Strauss, 2008) and cross‐case analysis (Miles and Huberman, 1994). We chose these methods for several reasons, and our choices reflect that this work straddles the boundaries between primary qualitative research and synthesis of systematic reviews. First, we used framework synthesis to structure our data extraction from included reviews by Toulmin's theory of argumentation, described in the Introduction. Framework synthesis is especially helpful when a large body of data could potentially inform an answer to a question, as it structures data extraction and organizes included data by using a priori labels and concepts (Oliver *et al*., 2008; Carroll *et al*., 2013). Given that our intention was to interpret and understand the argumentation in each review, we used grounded theory oriented in the Straussian method to develop and theorize the underlying ‘basic social/psychological processes’ (Corbin and Strauss, 2008), or, as we labelled them the ‘modes of reasoning’, inherent in meta‐analysis and narrative synthesis. These modes of reasoning became our ‘core categories’ or the organizing processes that emerge from a grounded theoretical approach to qualitative analysis. As our grounded theory findings developed, we used cross‐case analysis to understand how these modes of reasoning were distinct and overlapping.

### 2.2 Analysis and synthesis

For each included review, we searched for and extracted four pieces of information. First, we looked for stated rationales for undertaking, or not undertaking, a specific synthesis method. Second, we looked for a description of the synthesis methods reported, that is, how authors ‘put the studies together’. For meta‐analytic syntheses, this was often the detail of statistical analysis, and for narrative synthesis, this was the use of an evidence rating scheme or description as to how studies were assembled into a synthesis. Third, we looked for what we described as the ‘synthetic warrant’, or the section of the results that captured the synthesis of intervention effects. Finally, we looked for the ‘synthetic claim’, or the section of the Discussion and Conclusion that offered an interpretation of the synthesis arising from the warrant. Consistent with the framework approach, these data were placed against the codes to ‘bring order’ to the large volume of data while maintaining each piece of data from the systematic reviews in context (Ritchie and Spencer, 1994). As data extraction proceeded, we inspected the data to understand relationships between the different components. This followed the Straussian grounded theory process of clustering specific close observations about the data (open codes) into axial codes or codes that describe characteristics of the phenomenon under investigation (Heath and Cowley, 2004; Corbin and Strauss, 2008; File S1). These axial codes then formed the properties of the core category (i.e. aspects of the core category that characterize it) or, as we labelled them, ‘modes of reasoning’, that emerged for each type of synthesis method. We used cross‐case analysis to compare the properties for each of the categories and to illuminate the differences between types of synthesis (Miles and Huberman, 1994). Analysis was led by the first author, who was responsible for extraction and coding.

Throughout the analysis, the lead author conferenced key themes and ideas based on the data with the wider team of researchers as a form of audit and transparency check. All researchers involved in this project had experience of systematic reviewing across a variety of topics and methods. Because the goal of this analysis was to examine rationales and logical processes as presented on the page, we did not contact the authors, as we believed that this would add extraneous information.

## 3 Results

In the 85 included systematic reviews, we analysed statements related to narrative synthesis in 67 of them and statements related to meta‐analysis (or pre‐planned but unexecuted meta‐analysis) in 20 of them. Two systematic reviews overlapped. Together, the systematic reviews covered a diversity of overlapping intervention domains (Table 2). Nearly a third of systematic reviews (31%) focused on physical activity, 20% focused on nutrition, and 15% of included reviews included a diversity of intervention domains (i.e. they did not necessarily set out to restrict by type of intervention). Details of included syntheses are in File S1.

**Table 2:** Characteristics of included systematic reviews

Characteristic	*n* (%)
Synthesis type	
Narrative synthesis	67 (79)
Meta‐analysis	20 (24)
Intervention domain	
Breastfeeding	1 (1)
Comprehensive	5 (6)
CVD prevention	4 (5)
Diverse	13 (15)
Mental health	12 (14)
Musculoskeletal symptoms	7 (8)
Nutrition	17 (20)
Physical activity	26 (31)
Policy and scheduling	6 (7)
Sexual health	2 (2)
Smoking cessation	7 (8)

### 3.1 Practical–configurational and inferential–predictive modes of reasoning

Throughout our analysis, the two core categories that emerged to frame the contrast between narrative synthesis and meta‐analysis were the practical–configurational mode of reasoning in narrative synthesis and the inferential–predictive mode of reasoning in meta‐analysis (Table 3). Practical–configurational reasoning focuses on making sense of the *reading* of the evidence – ‘what is going on here?’ or ‘what picture emerges?’ In contrast, inferential–predictive reasoning focuses on estimating the effectiveness of the intervention and potentially exploring heterogeneity in effectiveness – ‘does it work, and how well?’ and ‘will it work again?’

**Table 3:** Distinctions between modes of reasoning

Practical–configurational reasoning in narrative syntheses	Inferential–predictive reasoning in meta‐analysis
What is going on here? What picture emerges?	Does it work, and how well? Will it work again?
Warrants for the synthesis are implicit, or syntheses are both warrant and claim.	Warrant is the pooled effect size and confidence interval; claim is the interpretation.
Quality and consistency as part of the warrant and claim	Quality as a qualifier of the claim, consistency (including heterogeneity indices and confidence intervals) as both qualifier and additional claims
Grading systems render practical–configurational reasoning transparent.	Grading systems and summary statements qualify claims.

### 3.2 Warrant–claim distinctions

One way in which the distinction between modes of reasoning made itself apparent was the difference in warrant–claim distinctions between narrative synthesis and meta‐analysis. In papers that reported meta‐analyses, the Results section consisted of the reporting of the standard systematic review processes (search and flow of studies) alongside a pooled effect size with confidence interval and a heterogeneity index. The conclusions then included a statement interpreting the pooled effect size and making a claim about the included studies. That is to say, the pooled effect size was the key inferential warrant that linked the included studies to the final interpretation (the claim), which placed the effect size in context of its predictive value for future public health intervention. For example, Anderson *et al*. (2009) conducted a systematic review of worksite nutrition and physical activity interventions. They included six randomized controlled trials in their analysis of body mass index reductions. In the results section of their paper, they write that ‘the pooled effect at the 6–12‐month follow‐up was −0.47 BMI units (95% CI −0.75, −0.19) in favour of the intervention group’. In the Discussion section, they return to this pooled effect size to note that the review showed ‘evidence of a modest reduction in weight as a result of worksite health promotion programmes aimed at improving nutrition, physical activity, or both’. Similarly, confidence intervals were a warrant; that is, confidence intervals served to support the claim of an effect, or that an effect was not in evidence. Confidence intervals served as warrants both by showing the plausible distribution of effects and by demonstrating statistical significance.

By comparison, we often found it difficult to establish a distinction between warrant and claim in narrative syntheses and our preconception of narrative synthesis, like statistical pooling, as ‘bridging’ between data and interpretation in many cases broke down when applied to the systematic reviews that we analysed. In many of the narrative syntheses that we analysed, the findings sections consisted of a tabulation and summary of characteristics of included studies. These characteristics frequently included types or modality of intervention, intervention components or intervention setting or context; in cases where interventions were homogeneous in design, tabulation included characteristics such as study design and date range undertaken. This raises two possibilities: First, that warrants in narrative synthesis are implicit, and second, that the synthesis is both warrant and claim. As an example of how warrants are implicit, Bambra *et al*.'s (2008) review on compressed working weeks for worker health and wellbeing is emblematic. They summarize which studies showed positive (statistically significant evidence of improvement), negative (statistically significant evidence of worsening) and no effects, and then note as the synthesis that ‘the evidence base on the health effects of [compressed working week] interventions is perhaps best described as cautiously positive’, without specifically considering magnitude of effect. They cite the basis of the five highest quality studies having consistent effects as one reason (but it does not appear the only one) for this finding, although the totality of their reasoning remains implicit.

In the second case, presented syntheses could be viewed as being both warrant and claim because narrative synthesis brings to the foreground the configurational aspect of synthesis; that is, the sorting and sense making that goes into bringing order, describing intervention, context and outcome patterns and preparing studies for presentation is itself a synthesis. For example, in their systematic review of workplace interventions for alcohol misuse, Webb *et al*. (2009) prioritize the classification and description of different approaches to intervention. They located ten studies. In an example of this sorting and sense making, they noted that workplace alcohol interventions consisted of ‘three broad types of strategies: psychosocial skills training […]; brief intervention, including feedback of results of self‐reported drinking, life‐style factors and general health checks […]; and alcohol education delivered via an Internet website…’. They subsequently conclude that while differences between studies made a synoptic view of the evidence impossible, ‘brief interventions, interventions contained within health and life‐style checks, psychosocial skills training and peer referral may have potential to produce beneficial results’. Although they present a general narrative of effectiveness of the interventions, highlighting that interventions generally produced positive effects, they do not justify this specific claim about effectiveness with an explicit warrant, preferring instead to focus on agenda setting.

### 3.3 The role of quality and consistency in argumentation

Another distinction emerged in the role of consistency and quality of evidence in narrative synthesis as opposed to meta‐analysis.

In meta‐analyses, quality of the evidence was a qualifier of the claim advanced through the synthesis, while consistency could act as both a qualifier and a generator of additional claims to be supported through warrants. Quality was usually assessed at two stages of the review: through quality appraisal or risk of bias of the individual primary studies and through robustness checks or risk of bias across the studies (e.g. sensitivity analyses and tests for publication bias). That is, quality is considered both within studies and across the synthesis. For example, in their systematic review of interventions for preventing obesity in adults, Kremers *et al*. (2010) note that for the outcome of reductions in body mass index, the pooled effect size was statistically significant but ‘small, and it is important to recognize that publication bias is likely to favour interventions that show positive impacts’. In this example, the claim is qualified by drawing attention to the risk of bias across the dataset due to the potential for publication bias.

Consistency was both a qualifier and a generator of additional claims. In meta‐analysis, consistency in terms of the results of the primary studies (i.e., are the intervention effects all in the same direction or not and of similar magnitude) is tested through heterogeneity analyses. This highlights another role for confidence intervals as not only warrants but also as qualifiers when they demonstrate heterogeneity and uncertainty. The presence of heterogeneity (thereby indicating a lack of consistency) was varyingly portrayed as a limitation (e.g. of small sample sizes) or as something to be modelled and explained – i.e. a useful piece of information to help with the inferential aim underpinning the meta‐analysis. Both views of heterogeneity could occur in the same analysis. Where heterogeneity was seen as informative, it was both a rebuttal for the claim with implications for future inference and constitutive of a warrant and claim in its own right. For example, Kremers *et al*. (2010) used meta‐regression to demonstrate that the difference between interventions targeting weight management and interventions not targeting weight management was significant and favoured weight management‐targeted interventions. They subsequently note that ‘our quantitative analyses did show the importance of formulating the programme goal specifically towards weight management. Those interventions were found to be more successful […]’. This statement rebuts a claim of general effectiveness by restricting it to weight management‐targeted interventions.

In contrast, narrative syntheses placed consistency and quality of effect in the middle of the synthesis; that is, consistency (often implied to mean similarity in direction and magnitude of effects across included studies) and quality were also part of the warrant and the claim rather than qualifiers of it. This characterizes practical–configurational reasoning in that the combined ‘warrant–claim’ arising from narrative syntheses considers intervention effectiveness, consistency and quality as part of one synthetic statement of the evidence, generally of the sort that would be included in a practice guideline. For example, in a systematic review about workplace interventions for prevention of back pain, Bigos *et al*. (2009) state that ‘strong evidence (consistent findings in multiple, high‐quality trials) supports the following about preventing episodes of [back pain]: (1) Exercise programmes are effective, and 2) other interventions are not effective […]’. In other words, the link between the warrant and the claim is typically made directly by considering the consistency and quality of the evidence, rather than as is the case in meta‐analysis, where consistency and quality moderate the claim in some way.

### 3.4 Qualifying synthetic claims with grading systems

Narrative syntheses often used various devices to make their syntheses open and transparent. In narrative syntheses, systematic reviewers deployed a broad range of evidence grading rules to create the narrative synthesis, that is, as backings to support often implicit warrants. These rules reflected a configurational mode of reasoning because they were applied only to specific interventional subgroups that reviewers identified and followed a practical mode of reasoning by integrating consistency and quality of evidence in the middle of the synthesis. Systematic reviewers often cited methodological precursors, such as the former Cochrane Back Group's (now Cochrane Neck and Back Group) evidence grading rules (van Tulder *et al*., 2003) as a backing in their argumentation.

In contrast, systematic reviews with meta‐analyses often drew on summary statements of the quality of the evidence in contextualizing and interpreting their findings as qualifiers (both positive and negative) for the claim made from the meta‐analytic warrant. In only a few cases, this was by use of explicit grading systems, such as the *Community Guide* tool (Briss *et al*., 2000) in Anderson *et al*.'s (2009) meta‐analytic review mentioned in the preceding texts or Grading of Recommendations Assessment, Development and Evaluation (Guyatt *et al*., 2008) in Verweij *et al*.'s (2011) meta‐analysis on workplace physical activity interventions. But unlike in most narrative syntheses, these qualifiers that systematic reviewers made alongside meta‐analyses were not transparent in respect of the tools or other guidance used.

For example, many claims based on meta‐analyses qualified the evidence as being ‘strong’ or ‘weak’ without clarifying how appraisal of individual trials was transformed into the qualifier for the synthetic claim. In Tan *et al*.'s (2014) review of workplace interventions for prevention of depression, they note how they critically appraised included trials by using a checklist, and then conclude that evidence was of ‘good quality’ in support of the effectiveness of cognitive‐behavioural interventions. The use of ‘sense of the evidence’ statements as qualifiers could be viewed as evidence of practical–configurational reasoning in making sense of quality of evidence, although in this case, evidence grading comes at the end of the synthesis, rather than in the middle.

### 3.5 Presented rationales for synthesis methods

About half of the narrative syntheses we examined presented specific rationales for not undertaking meta‐analysis, all to do with heterogeneity in interventions and study features, and about half of the meta‐analyses we examined presented the use of statistical pooling methods as a distinct advantage of the systematic review.

## 4 Discussion

Although methods for systematic reviews of intervention effectiveness may regard narrative synthesis as a second‐choice option to meta‐analysis, our findings suggest that the modes of reasoning employed in each type of synthesis are dissimilar. In this analysis, we described the difference between narrative synthesis and meta‐analysis as being between practical–configurational modes of reasoning and inferential–predictive modes of reasoning. These modes of reasoning are distinct in the way they ‘use’ the included studies to synthesize evidence and, thus, construct an argument based on the included studies. These distinctions are especially clear in the warrant–claim distinction and the roles of consistency and quality, but these modes of reasoning do not exist independently. Given the large number of narrative syntheses that explained why meta‐analysis was not pursued, it was clear in narrative syntheses that the ‘frame of reference’ was one of meta‐analysis, and similarly, in meta‐analyses, elements of practical–configurational reasoning were implicit.

### 4.1 Implications

The implications of these findings are both sociological and methodological. Sociologically, we know relatively little about the ‘machinery’ of systematic reviewing. Moreira's (2007) ethnographic study of systematic review groups identified the key processes of ‘disentanglement and qualification’, in which information from studies is located and removed from the texts where it is found and then reassessed by using a variety of tools to form a synthesis. Shepherd's (2013) interview‐based study of systematic reviewers working in health promotion identified that the reviewers had a difficult time making explicit their understandings of how to synthesize studies, noting as well that judgment is key in appraising studies. Our analysis here extends upon the work of both studies by deploying argumentation theory to examine the modes of reasoning around syntheses as presented on the page as part of the finished product. On the one hand, we draw distinctions between how systematic reviewers represent different types of ‘qualification’ of the data. On the other hand, we also show how judgment is present throughout the presentation of the processes of synthesis, regardless of whether the synthesis method is narrative or meta‐analytic.

Although our analysis focuses on the synthesis aspect of reviews, our findings do also have implications for how we consider bias in the context of review processes more broadly. Assertions in method guidance that narrative syntheses may be more subject to bias (Reeves *et al*., 2011) focus on one aspect of the review to the exclusion of other potential reviewer‐level sources of bias. In addition to the informal or formal evidence grading systems used in meta‐analyses, risk of bias rating schemes and in reviews of complex interventions especially, decisions of which evaluations include the target class of interventions of interest require extensive judgment. Empirical investigations of how much ‘judgment’ is embedded in risk of bias rating tools reveal poor inter‐rater reliability, both for tools used to assess observational designs (Hartling *et al*., 2013) and the Cochrane risk of bias tool (Hartling *et al*., 2013). While these findings are not an excuse for lack of rigour, they do highlight the importance of auditability and transparency in decision‐making.

From a methodological perspective, these findings complement existing guidance on how to undertake narrative syntheses (Popay *et al*., 2006) and on the role of configurational approaches in systematic reviews generally (Gough *et al*., 2012). Specifically, in systematic reviews where meta‐analysis is considered the primary synthesis method, parallel claims about the evidence arising from a complementary narrative synthesis require an auditable and transparent framework. One way of doing this is to employ pre‐specified grading systems, such as Grading of Recommendations Assessment, Development and Evaluation (Guyatt *et al*., 2008). Regardless of the synthesis method, one way that reviewers should ‘assess the robustness of their synthesis’ (Popay *et al*., 2006) is by reporting their practical–configurational reasoning in a clear and direct manner. It is also possible that narrative syntheses and meta‐analyses should not be judged on the same quality criteria for evaluating the soundness of a particular synthesis. This is because narrative syntheses display sense making in a very different way than meta‐analyses do. Thus, whereas examination of heterogeneity in meta‐analysis should ideally be pre‐specified (and thus post hoc analyses may be less credible), a narrative synthesis should seek to illuminate ways in which the evidence can be understood beyond a pooled effect size.

It is also worth considering how the links between warrant and claim are established and/or moderated in the two synthesis types. The different uses of information about consistency and quality in particular suggest that the reviewers are likely to be addressing research questions in different ways when using the different approaches. Past examinations of databases of systematic reviews have suggested that narrative syntheses are more likely to reach negative conclusions (Petticrew *et al*., 1999). This could be because the different approaches to argumentation suggest views of the evidence that differ based on the consideration of consistency and quality. Across methods and modes of reasoning, there is a general need – one that likely exists for systematic reviews universally – to focus less on statistical significance and more on confidence intervals as a marker of consistency and uncertainty. Although confidence intervals indicate whether or not a pooled intervention effect falls on the right side of the ‘bright line’ of 0.05, they are more informative about the pooled effect size *per se*.

Users of both methods should also consider how the question being explored or tested by their analyses might differ from the a priori intended questions – particularly in the case where the original method of choice was not eventually used. By corollary, while the ‘answers’ or claims may appear to be similar in semantic content between the different methods, they are the products of different questions and correspondingly, different modes of reasoning. However, as we demonstrated, there is some ‘bleed‐through’ between these modes of reasoning as well. The end goal of a meta‐analysis is not simply to produce a summary pooled effect size: Meta‐analysis can and should be used to explore the consistency of effect across a body of literature. Often, meta‐analytical techniques are abandoned due to ‘heterogeneity’ between studies, when in fact, meta‐analysis is possible and could yield useful results even if the pooling of effect sizes is not appropriate (Ioannidis *et al*., 2008). When narrative synthesis is employed as a fall‐back position, rather than as a complement to meta‐analysis, it may be the case that the question being explored or tested differs from the a priori intended questions. Where it is not possible to address the original review question, reviewers should be explicit in re‐framing the questions so that any claims for the evidence are clearly supported by warrants – i.e. they should be clear when deploying both claims and warrants that their focus has shifted from testing a statistical hypothesis to exploring context or patterns.

This ‘question drift’ between a priori intended questions and the questions answered by a specific synthesis method may also have implications for the downstream use of reviews to inform policy or practice. That is, the ‘messaging’ of the synthesis method used could influence the ability of reviews to be taken as evidence to inform decision‐making. As noted in the preceding texts, early methodological investigation showed that narrative syntheses may be more likely to reach negative conclusions (Petticrew *et al*., 1999). Yet, even when narrative syntheses reach positive conclusions, the decision to not undertake meta‐analysis may present the image that evidence is not ready for policy and practice, even when included studies collectively point to effectiveness of an intervention. To illustrate this point, consider a hypothetical systematic review of effectiveness of a public health intervention with a convincing number of adequately powered, high‐quality studies, each of which indicates a clinically and statistically significant effect of the intervention. If each evaluation uses different statistical methods, summary effect sizes and designs to evaluate the intervention, then the results may not be meaningfully pooled in a meta‐analysis, even if collective evidence suggests that an intervention has strong evidence of efficacy. On the other side, careful and considered use of a narrative synthesis method may be essential to illuminate the equity consequences of an intervention in a way that a subgroup analysis may not convincingly demonstrate. For example, a ‘small’ differential in effect on the proximal outcome in a population‐level public health intervention may have major downstream consequences for the health and wellbeing of subgroups that may not be highlighted by a meta‐analysis.

Finally, these findings may have relevance in systematic reviews beyond effectiveness and in applications beyond the specific appraisal of claims advanced in individual systematic reviews. The first implication is especially in areas such as qualitative meta‐syntheses, where ‘authorial privilege’ is an expected part of analysis. For example, recent work in systematizing and appraising confidence in the findings expressed in systematic reviews of qualitative research (CERQual; see Lewin *et al*., 2015) has shown the importance of rendering transparent ‘expert’ methods of reasoning across evidence. The second implication has relevance for the role of machine learning in generating evidence synthesis. Currently, uses of artificial intelligence to generate summaries of the evidence rely on the evaluation of ‘arguments’ postulated by individual reviews and trials (Hunter and Williams, 2012). While the use of ‘argument’ is a mathematical/logical construct in their application, it is clear from our work that there exists a diversity of arguments advanced in reviews. This work can help in a more nuanced understanding of the form and function of arguments advanced in different types of review.

### 4.2 Strengths and limitations

This meta‐research study was unusual in that it deployed qualitative methodology to analyse the methods and findings expressed in a subset of reviews from a larger overview of reviews. While this is a strength, it is also a limitation in that we had limited data on how syntheses proceeded to analyse from each paper, and thus limited depth to investigate how researchers actually applied the modes of reasoning that we have described. To a degree, we were able to substitute depth in individual papers for breadth across a large sample of studies. However, future studies could explore argumentation in methods in greater depth by examining specific review teams' work in an effort to understand how they arrived at their syntheses.

Our sampling frame was taken from an overview of systematic reviews in workplace health promotion (Brunton *et al*. 2016; relevant information in File S1). This had the benefit of identifying a coherent set of reviews that still presented considerable diversity in methods and outcomes. However, our findings may not be generalizable to other areas, for example, pharmacological interventions and population‐level or structural interventions. Future studies may wish to examine how modes of reasoning differ across different types of intervention.

In addition, as with studies conducted with any interpretive methodology, it is possible that an alternative analyst, or set of analysts, would have arrived at different conclusions than our group did. Our findings benefited from rigorous team audit to ensure a shared understanding of the phenomena at hand.

### 4.3 Conclusion

Narrative syntheses and meta‐analyses represent different modes of reasoning. To continue to advance systematic review methodology, it is important to consider narrative synthesis in its own right as a method and to develop specific quality criteria and understandings of how it is carried out, not merely as a complement to, or second‐best option for, meta‐analysis.

## Supporting information

Supporting info item

## References

[jrsm1231-bib-0001] Achana F , et al. 2014 An exploration of synthesis methods in public health evaluations of interventions concludes that the use of modern statistical methods would be beneficial. Journal of Clinical Epidemiology 67: 376–390. DOI:10.1016/j.jclinepi.2013.09.018. 24388291 10.1016/j.jclinepi.2013.09.018

[jrsm1231-bib-0002] Anderson, LM , et al. 2009 The effectiveness of worksite nutrition and physical activity interventions for controlling employee overweight and obesity. American Journal of Preventive Medicine 37: 340–357. Available at: http://linkinghub.elsevier.com/retrieve/pii/S0749379709004863 [Accessed October 19, 2015]. 19765507 10.1016/j.amepre.2009.07.003

[jrsm1231-bib-0003] Balgopal MM , Wallace AM , Dahlberg S . 2016 Writing from different cultural contexts: how college students frame an environmental SSI through written arguments. Journal of Research in Science Teaching (September). DOI:10.1002/tea.21342.

[jrsm1231-bib-0004] Bambra C et al. 2008 “A hard day's night?” The effects of compressed working week interventions on the health and work‐life balance of shift workers: a systematic review. Journal of Epidemiology and Community Health 62: 764–777. 18701725 10.1136/jech.2007.067249

[jrsm1231-bib-0005] Bigos SJ , et al. 2009 High‐quality controlled trials on preventing episodes of back problems: systematic literature review in working‐age adults. Spine Journal 9: 147–168. DOI:10.1016/j.spinee.2008.11.001. 19185272 10.1016/j.spinee.2008.11.001

[jrsm1231-bib-0006] Borenstein M et al. 2009 Introduction to Meta‐Analysis. Chichester, UK: John Wiley & Sons.

[jrsm1231-bib-0007] Briss PA , et al. 2000 Developing an evidence‐based guide to community preventive services—methods. American Journal of Preventive Medicine 18: 35–43. Available at: http://www.sciencedirect.com/science/article/pii/S0749379799001191 [Accessed October 19, 2015]. 10806978 10.1016/s0749-3797(99)00119-1

[jrsm1231-bib-0040] Brunton G , Dickson K , Khatwa M , Caird J , Oliver S , Hinds K , Thomas J . 2016 Developing Evidence‐Informed, Employer‐Led Workplace Health. London: EPPI‐Centre, Social Science Research Unit, UCL Institute of Education, University College London.

[jrsm1231-bib-0008] Carroll C , et al. 2013 “Best fit” framework synthesis: refining the method. BMC Medical Research Methodology 13: 37 Available at: http://www.pubmedcentral.nih.gov/articlerender.fcgi?artid=3618126&tool=pmcentrez&rendertype=abstract [Accessed October 19, 2015]. 23497061 10.1186/1471-2288-13-37 PMC3618126

[jrsm1231-bib-0009] Corbin JC , Strauss AL . 2008 Basics of Qualitative Research: Techniques and Procedures for Developing Grounded Theory. 3rd edn. Thousand Oaks, CA, US: Sage Publications.

[jrsm1231-bib-0010] Craig R . 1999 Communication theory as a field. Communication Theory 9: 119–161.

[jrsm1231-bib-0011] Gough D , Oliver S , Thomas J . 2012 An Introduction to Systematic Reviews. London: Sage.

[jrsm1231-bib-0012] Gray R , Kang N‐H . 2012 The structure of scientific arguments by secondary science teachers: comparison of experimental and historical science topics. International Journal of Science Education 36: 1–20. DOI:10.1080/09500693.2012.715779.

[jrsm1231-bib-0013] Guyatt GH , et al. 2008 GRADE: an emerging consensus on rating quality of evidence and strength of recommendations. BMJ 336: 924–926. Available at: http://www.pubmedcentral.nih.gov/articlerender.fcgi?artid=2335261&tool=pmcentrez&rendertype=abstract [Accessed October 19, 2015]. 18436948 10.1136/bmj.39489.470347.AD PMC2335261

[jrsm1231-bib-0014] Hartling L , Milne A , et al. 2013 Testing the Newcastle Ottawa Scale showed low reliability between individual reviewers. Journal of Clinical Epidemiology 66: 982–993. DOI:10.1016/j.jclinepi.2013.03.003. 23683848 10.1016/j.jclinepi.2013.03.003

[jrsm1231-bib-0015] Hartling L , Hamm MP , et al. 2013 Testing the risk of bias tool showed low reliability between individual reviewers and across consensus assessments of reviewer pairs. Journal of Clinical Epidemiology 66: 973–981. DOI:10.1016/j.jclinepi.2012.07.005. 22981249 10.1016/j.jclinepi.2012.07.005

[jrsm1231-bib-0016] Heath H , Cowley S . 2004 Developing a grounded theory approach: a comparison of Glaser and Strauss. International Journal of Nursing Studies 41: 141–150. Available at: http://linkinghub.elsevier.com/retrieve/pii/S0020748903001135 [Accessed October 9, 2012]. 14725778 10.1016/s0020-7489(03)00113-5

[jrsm1231-bib-0017] Hedges LV , Olkin I . 1980 Vote‐counting methods in research synthesis. Psychological Bulletin 88: 359–369.

[jrsm1231-bib-0018] Hunter A , Williams M . 2012 Aggregating evidence about the positive and negative effects of treatments. Artificial Intelligence in Medicine 56: 173–190. DOI:10.1016/j.artmed.2012.09.004. 23178172 10.1016/j.artmed.2012.09.004

[jrsm1231-bib-0019] Ioannidis JPA , Patsopoulos NA , Rothstein HR . 2008 Reasons or excuses for avoiding meta‐analysis in forest plots. BMJ 336: 1413–1415. 18566080 10.1136/bmj.a117 PMC2432114

[jrsm1231-bib-0020] Kremers S , et al. 2010 Systematic prevention of overweight and obesity in adults: a qualitative and quantitative literature analysis. Obesity Reviews 11: 371–379. DOI:10.1111/j.1467‐789X.2009.00598.x. 19538441 10.1111/j.1467-789X.2009.00598.x

[jrsm1231-bib-0021] Labrie N , Schulz PJ . 2014 Does argumentation matter? A systematic literature review on the role of argumentation in doctor‐patient communication. Health Communication 29: 996–1008. Available at: http://www.ncbi.nlm.nih.gov/pubmed/24359318 [Accessed October 19, 2015]. 24359318 10.1080/10410236.2013.829018

[jrsm1231-bib-0022] Lewin S , et al. 2015 Using qualitative evidence in decision making for health and social interventions: an approach to assess confidence in findings from qualitative evidence syntheses (GRADE‐CERQual). PLoS Medicine 12: e1001895 Available at: http://journals.plos.org/plosmedicine/article?id=10.1371/journal.pmed.1001895 [Accessed October 19, 2015]. 26506244 10.1371/journal.pmed.1001895 PMC4624425

[jrsm1231-bib-0023] Miles MB , Huberman AM . 1994 Qualitative Data Analysis: An Expanded Sourcebook. 2nd edn. Thousand Oaks, CA: Sage Publications.

[jrsm1231-bib-0024] Moreira T . 2007 Entangled evidence: knowledge making in systematic reviews in healthcare. Sociology of Health & Illness 29: 180–197. DOI:10.1111/j.1467‐9566.2007.00531.x. 17381812 10.1111/j.1467-9566.2007.00531.x

[jrsm1231-bib-0025] Oliver SR et al. 2008 A multidimensional conceptual framework for analysing public involvement in health services research. Health Expectations 11: 72–84. 18275404 10.1111/j.1369-7625.2007.00476.x PMC5060424

[jrsm1231-bib-0026] Petticrew M , Anderson L , et al. 2013 Complex interventions and their implications for systematic reviews: a pragmatic approach. Journal of Clinical Epidemiology 66: 1209–1214. Available at: http://www.ncbi.nlm.nih.gov/pubmed/23953085 [Accessed September 16, 2014]. 23953085 10.1016/j.jclinepi.2013.06.004

[jrsm1231-bib-0027] Petticrew M , et al. 1999 Quality‐assessed reviews of health care interventions and the database of abstracts of reviews of effectiveness (DARE). NHS CRD Review, Dissemination, and Information Teams. International Journal of Technology Assessment in Health Care 15: 671–678. Available at: http://www.mrw.interscience.wiley.com/cochrane/clcmr/articles/CMR‐4428/frame.html [Accessed October 19, 2015]. 10645108

[jrsm1231-bib-0028] Petticrew M , Rehfuess E , et al. 2013 Synthesizing evidence on complex interventions: how meta‐analytical, qualitative, and mixed‐method approaches can contribute. Journal of Clinical Epidemiology 66: 1230–1243. Available at: http://www.ncbi.nlm.nih.gov/pubmed/23953082 [Accessed August 28, 2014]. 23953082 10.1016/j.jclinepi.2013.06.005

[jrsm1231-bib-0029] Petticrew M , Roberts H . 2006 Systematic Reviews in the Social Sciences: A Practical Guide. Malden, MA: Blackwell Publishing.

[jrsm1231-bib-0030] Popay J et al. 2006 Guidance on the Conduct of Narrative Synthesis in Systematic Reviews: A Product from the ESRC Methods Programme. Lancaster, UK.

[jrsm1231-bib-0031] Reeves BC et al. 2011 Chapter 13: Including non‐randomized studies In Higgins JPT, Green S (eds.). Cochrane Handbook for Systematic Reviews of Interventions. The Cochrane Collaboration.

[jrsm1231-bib-0032] Ritchie J , Spencer L . 1994 Qualitative data analysis for applied policy research In Analysing Qualitative Data. London: Routledge, pp. 173–194.

[jrsm1231-bib-0033] Shepherd J . 2013 Judgment, resources, and complexity: a qualitative study of the experiences of systematic reviewers of health promotion. Evaluation & the Health Professions 36: 247–267. 22615497 10.1177/0163278712447222

[jrsm1231-bib-0034] Tan L , et al. 2014 Preventing the development of depression at work: a systematic review and meta‐analysis of universal interventions in the workplace. BMC Medicine 12: 74 Available at: http://www.pubmedcentral.nih.gov/articlerender.fcgi?artid=4014627&tool=pmcentrez&rendertype=abstract [Accessed October 19, 2015]. 24886246 10.1186/1741-7015-12-74 PMC4014627

[jrsm1231-bib-0035] Toulmin S . 2003 The Uses of Argument. 2nd edn. Cambridge, UK: Cambridge University Press.

[jrsm1231-bib-0036] van Tulder M , et al. 2003 Updated method guidelines for systematic reviews in the cochrane collaboration back review group. Spine 28: 1290–1299. Available at: http://content.wkhealth.com/linkback/openurl?sid=WKPTLP:landingpage&an=00007632‐200306150‐00014 [Accessed October 19, 2015]. 12811274 10.1097/01.BRS.0000065484.95996.AF

[jrsm1231-bib-0037] Verweij LM , et al. 2011 Meta‐analyses of workplace physical activity and dietary behaviour interventions on weight outcomes. Obesity Reviews 12: 406–429. DOI:10.1111/j.1467‐789X.2010.00765.x. 20546142 10.1111/j.1467-789X.2010.00765.x

[jrsm1231-bib-0038] Webb G , et al. 2009 A systematic review of work‐place interventions for alcohol‐related problems. Addiction 104: 365–377. DOI:10.1111/j.1360‐0443.2008.02472.x. 19207344 10.1111/j.1360-0443.2008.02472.x

[jrsm1231-bib-0039] Whithaus C . 2012 Claim‐Evidence Structures in Environmental Science Writing: Modifying Toulmin's Model to Account for Multimodal Arguments. Technical Communication Quarterly 21: 105–128.

