# Supplementary material for: Interpretive analysis of 85 systematic reviews suggests that narrative syntheses and meta‐analyses are incommensurate in argumentation
Source: Res Synth Methods. 2016 Nov 17;8(1):109–18. doi: 10.1002/jrsm.1231 (PMC5347877; doi:10.1002/jrsm.1231)
Supplement: Supplementary file 1 — Supporting info item [file JRSM-8-109-s001.docx]

**Supplementary File 1**

**Search and retrieval methods, additional coding and included systematic reviews**

**1. Search and retrieval methods**

**Searching**

**Sources.** Potentially relevant citations for a) systematic reviews, b) research on stakeholders’ views and c) policy documents, were located through a variety of sources including: contact with key informants, electronic citation database searches, and websites.

**Search strategy.** Searches were limited to citations published between 1995 and 2015. Search strings based on a combination of free-text and database-specific terms were developed in collaboration with our Information Scientist. The concepts combined included: (workplace terms) AND (systematic review terms). The search strategy was developed first in PubMed and then translated using syntax suitable for other databases. The PubMed search strategy is as follows:

("Workplace"[Mesh] OR (workplace[Title/Abstract] OR worksite[Title/Abstract] OR employer[Title/Abstract] OR employee[Title/Abstract] OR employees[Title/Abstract])) AND (("Review" [Publication Type] OR ("systematic review"[Title/Abstract] OR "meta-analysis"[Title/Abstract]))

Publication date from 1995/01/01 to 2015/12/31.

Located citations were uploaded into EPPI-Reviewer custom research software, for management of publication retrieval, coding and synthesis (Thomas et al. 2010).

***Key informants.*** Research commissioners were asked for any relevant reviews of effectiveness, research on stakeholder views about barriers or facilitators to successful implementation of workplace initiatives, and relevant policy documents discussing characteristics of successful workplace strategies.

***Electronic databases.*** The following electronic sources of systematic reviews were searched:

- MEDLINE
- DARE
- Cochrane Library
- PsycINFO
- Database of Promoting Health Effectiveness Reviews (DoPHER).

***Websites.*** Google Scholar and the King’s Fund website were searched for relevant publications. To locate policy documents that recommend or outline key characteristics of successful workplace health interventions, we searched 43 websites of health promoting organisations, bodies promoting corporate social responsibility and health departments of regional and national government websites of key workplace health organisations. These included:

- World Health Organization
- UK National Institute for Health and Care Excellence
- UK Health at Work Policy Unit
- The Canadian Centre for Occupational Health and Safety
- The US Centers for Disease Control
- The American College of Occupational and Environmental Medicine.

The majority of searching was conducted on UK websites but we visited a few government sites in the United States, Canada, Europe and Australia to make a comparison with the UK context. We also contacted a policy analyst on workplace health promotion at Public Health England to source documents. We excluded non-systematic reviews and case studies of organisations with recognised high quality workplace health promotion interventions.

**Screening for study inclusion/exclusion**

All located citations were assessed first on the basis of title and abstract. The full publication of those meeting inclusion criteria were retrieved and assessed again for inclusion.

**Eligibility criteria for systematic reviews.** For systematic review citations to be included on title and abstract, studies had to:

- be published from 1995 to present;
- be in English (although non-English studies were marked for future assessment as appropriate);
- describe the search strategy, inclusion criteria and quality assessment methods
- evaluate interventions delivered in a workplace setting; and
- report healthcare or wellbeing outcomes.

**Quality assurance**

To strengthen the application of these methods, quality assurance procedures were followed. Two reviewers developed searches in collaboration with our Information Scientist. Two reviewers screened the same selection of retrieved references, then met to cross-check their screening results and establish agreement on the use of inclusion and exclusion criteria. Once an inter-rater reliability of more than 90% was established, reviewers screened references independently. Disagreements or queries on inclusion were referred to a third reviewer as needed. The same quality assurance process was applied to the coding of studies. Two reviewers assessed reviews for risk of bias and met to discuss and agree ratings, with disagreements resolved by a third reviewer where necessary.

**2. Additional coding**

We document here the axial coding and aspects of the cross-case analysis that led to the formation of the two modes of reasoning that organised our analysis.

**Excerpts from axial coding**

| **Narrative syntheses** | **Meta-analyses** |
| --- | --- |
| Grading systems used instead of meta-analysis—‘alternative’ to meta-analysis?  Grading systems used to formalise reasoning  Grading systems reflecting a way of formalising ‘sense of the evidence’  Analyses oriented towards ‘sense of the evidence’  Cataloguing, categorising evidence  Bringing order to evidence—where is the field going?  Quality of evidence integral to interpretations and arguments  Quality of evidence as key conclusion  Warrants implicit in claims  How well the evidence ‘hangs together’ important in narrative reasoning  Evidence ‘hanging together’ defined by similarity of study outcomes—consistency  Analyses dominated by need to make sense of the evidence and understand in subgroups as appropriate | Grading systems ancillary to conclusion of effectiveness  Statements about strength of evidence generally ‘peripheral’ to conclusion; often unformalised  Evidence statements attenuated by quality  Claims focus on ‘does it work?’  Substantiate ‘does it work?’ with size of effect  How certain we are of effectiveness expressed by confidence intervals (and secondarily by p-values)  P-values—support vs. no support for effectiveness  Heterogeneity ‘attenuates’ conclusion  Heterogeneity opens up additional lines of enquiry, e.g. by meta-regression  Statistical pooling links included studies with ‘does it work?’  Analyses focusing on pooling to determine effectiveness, including generalizability |

**Cross-case analysis: consistency**

| **Narrative synthesis** | **Meta-analysis** |
| --- | --- |
| Consistency defined by reading of the evidence  Consistency as part of claim  Methods for determining consistency implicit | Consistency defined by heterogeneity and confidence intervals  Consistency as part of qualifier to claim and generator of additional claims  Consistency a ‘statistical’ process (I^2^, Cochran’s Q) |

**Cross-case analysis: grading systems and quality of evidence**

| **Narrative synthesis** | **Meta-analysis** |
| --- | --- |
| Grading systems substantiate otherwise implicit warrants  When grading systems are used, appeal to methodological precedent  Quality as key conclusion of the analysis | Grading systems used to qualify conclusions  Statements of quality of evidence often informal and unclear in basis  Quality as qualifier of conclusion and interpretation |

**3. Details of included syntheses**

| **Reference** | **Synthesis** | **Intervention domain** |
| --- | --- | --- |
| Abdulwadud OA, and Snow ME. (2012). Interventions in the workplace to support breastfeeding for women in employment. Cochrane Database of Systematic Reviews: Reviews, Issue 10, pp. | Narrative and meta-analysis | Breastfeeding |
| Abraham C, and Graham-Rowe E. (2009). Are worksite interventions effective in increasing physical activity? A systematic review and meta-analysis. Health Psychology Review, 3(1), pp.108-144. | Meta-analysis | Physical activity |
| Anderson LM, Quinn TA, Glanz K, Ramirez G, Kahwati LC, Johnson DB, Buchanan LR, Archer WR, Chattopadhyay S, Kalra GP, and Katz DL. (2009). The effectiveness of worksite nutrition and physical activity interventions for controlling employee overweight and obesity: a systematic review. American journal of preventive medicine, 37(4), pp.340-57. | Meta-analysis | Physical activity, nutrition |
| Aneni EC, Roberson LL, Maziak W, Agatston AS, Feldman T, Rouseff M, Tran TH, Blumenthal RS, Blaha MJ, Blankstein R, Al-Mallah MH, Budoff MJ, and Nasir K. (2014). A systematic review of internet-based worksite wellness approaches for cardiovascular disease risk management: outcomes, challenges & opportunities. PloS one, 9(1), pp.e83594. | Narrative | CVD prevention |
| Aust B, and Ducki A. (2004). Comprehensive health promotion interventions at the workplace: experiences with health circles in Germany. Journal of Occupational Health Psychology, 9(3), pp.258-270. | Narrative | Comprehensive |
| Bambra C, Whitehead M, Sowden A, Akers J, and Petticrew M. (2008). "A hard day's night?" The effects of Compressed Working Week interventions on the health and work-life balance of shift workers: a systematic review. Journal of Epidemiology and Community Health, 62(9), pp.764-777. | Narrative | Policy and scheduling |
| Bambra CL, Whitehead MM, Sowden AJ, Akers J, and Petticrew MP. (2008). Shifting schedules: the health effects of reorganizing shift work. American journal of preventive medicine, 34(5), pp.427-434. | Narrative | Policy and scheduling |
| Barr-Anderson DJ, Auyoung Mona, Whitt-Glover Melicia C, Glenn Beth A, and Yancey Antronette K. (2011). Integration of Short Bouts of Physical Activity Into Organizational Routine: A Systematic Review of the Literature. American Journal of Preventive Medicine, 40(1,), pp.76-93. | Narrative | Physical activity |
| Bell JA, and Burnett A. (2009). Exercise for the primary, secondary and tertiary prevention of low back pain in the workplace: a systematic review. Journal of occupational rehabilitation, 19(1), pp.8-24. | Narrative | Musculoskeletal symptoms |
| Bigos SJ, Holland J, Holland C, Webster JS, Battie M, and Malmgren JA. (2009). High-quality controlled trials on preventing episodes of back problems: systematic literature review in working-age adults. The spine journal : official journal of the North American Spine Society, 9(2), pp.147-68. | Narrative | Musculoskeletal symptoms |
| Boocock MG, McNair PJ, Larmer PJ, Armstrong B, Collier J, Simmonds M, and Garrett N. (2007). Interventions for the prevention and management of neck/upper extremity musculoskeletal conditions: a systematic review. Occupational and environmental medicine, 64(5), pp.291-303. | Narrative | Musculoskeletal symptoms |
| Brown HE, Gilson ND, Burton NW, and Brown WJ. (2011). Does physical activity impact on presenteeism and other indicators of workplace well-being?. Sports Medicine, 41(3), pp.249-262. | Narrative | Physical activity |
| Burnhams NH, Musekiwa A, Parry C, and London L. (2013). A systematic review of evidence-based workplace prevention programmes that address substance abuse and HIV risk behaviours. [online] Centre for Research and Information on Substance Abuse Nigeria. Available at: http://ovidsp.ovid.com/ovidweb.cgi?T=JS&PAGE=reference&D=psyc10&NEWS=N&AN=2013-40080-001. | Narrative | Sexual health, mental health and substance use |
| Cahill K, and Perera R. (2008). Competitions and incentives for smoking cessation. The Cochrane database of systematic reviews, (3), pp.CD004307. | Meta-analysis | Smoking cessation |
| Cahill K, Moher M, and Lancaster T. (2008). Workplace interventions for smoking cessation. The Cochrane database of systematic reviews, (4), pp.CD003440. | Meta-analysis | Smoking cessation |
| Cancelliere C, Cassidy JD, Ammendolia C, and Cote P. (2011). Are workplace health promotion programs effective at improving presenteeism in workers? A systematic review and best evidence synthesis of the literature. BMC public health, 11, pp.395. | Narrative | Diverse |
| Cassidy JD, and Cote P. (2008). Is it time for a population health approach to neck pain?. Journal of manipulative and physiological therapeutics, 31(6), pp.442-6. | Narrative | Musculoskeletal symptoms |
| Chan CW, and Perry L. (2012). Lifestyle health promotion interventions for the nursing workforce: a systematic review. Journal of clinical nursing, 21(15-16), pp.2247-61. | Narrative | Diverse |
| Chau JY, van der Ploeg HP, van Uffelen JGZ, Wong J, Riphagen I, Healy GN, Gilson ND, Dunstan DW, Bauman AE, Owen N, and Brown WJ. (2010). Are workplace interventions to reduce sitting effective? A systematic review. Preventive Medicine: An International Journal Devoted to Practice and Theory, 51(5), pp.352-356. | Narrative | Physical activity |
| Chu AH, Koh D, Moy FM, and Muller-Riemenschneider F. (2014). Do workplace physical activity interventions improve mental health outcomes?. Occupational Medicine, 64(4), pp.235-245. | Narrative | Mental health and substance use |
| Ciliska D, Robinson P, Armour T, Ellis P, Brouwers M, Gauld M, Baldassarre F, and Raina P. (2005). Diffusion and dissemination of evidence-based dietary strategies for the prevention of cancer. Nutrition journal, 4, pp.13. | Narrative | Nutrition |
| Compernolle S, De Cocker K, Lakerveld J, Mackenbach JD, Nijpels G, Oppert JM, Rutter H, Teixeira PJ, Cardon G, and De Bourdeaudhuij I. (2014). A RE-AIM evaluation of evidence-based multi-level interventions to improve obesity-related behaviours in adults: a systematic review (the SPOTLIGHT project). The international journal of behavioral nutrition and physical activity, 11(1), pp.147. | Narrative | Physical activity, nutrition |
| Conn VS, Hafdahl AR, Cooper PS, Brown LM, and Lusk SL. (2009). Meta-analysis of workplace physical activity interventions. American journal of preventive medicine, 37(4), pp.330-9. | Meta-analysis | Physical activity |
| Crawford JO, Graveling RA, Cowie HA, and Dixon K. (2010). The health safety and health promotion needs of older workers. Occupational medicine (Oxford, and England), 60(3), pp.184-92. | Narrative | Diverse |
| Dietrich S, Deckert S, Ceynowa M, Hegerl U, and Stengler K. (2012). Depression in the workplace: a systematic review of evidence-based prevention strategies. International archives of occupational and environmental health, 85(1), pp.1-11. | Narrative | Mental health and substance use |
| Dishman R K, Oldenburg B, O’Neal H, and Shephard R J. (1998). Worksite physical activity interventions. American Journal of Preventive Medicine, 15, pp.344-61. | Meta-analysis | Physical activity |
| Engbers L H, van Poppel M N, Chin A Paw M J, and van Mechelen W. (2005). Worksite health promotion programs with environmental changes: a systematic review. American Journal of Preventive Medicine, 29(1), pp.61-70. | Narrative | Physical activity, nutrition |
| Freak-Poli RLA, Cumpston M, Peeters A, and Clemes SA. (2013). Workplace pedometer interventions for increasing physical activity. Cochrane Database of Systematic Reviews: Reviews, Issue 4, pp. | Meta-analysis | Physical activity |
| Furlan AD, Gnam WH, Carnide N, Irvin E, Amick BC, DeRango K, McMaster R, Cullen K, Slack T, Brouwer S, and Bultmann U. (2012). Systematic review of intervention practices for depression in the workplace. [online] Springer Germany. Available at: http://ovidsp.ovid.com/ovidweb.cgi?T=JS&PAGE=reference&D=psyc9&NEWS=N&AN=2012-20781-004. | Narrative | Mental health and substance use |
| Geaney F, Kelly C, Greiner BA, Harrington JM, Perry IJ, and Beirne P. (2013). The effectiveness of workplace dietary modification interventions: a systematic review. Preventive medicine, 57(5), pp.438-47. | Narrative | Nutrition |
| Graveling RA, Crawford JO, Cowie H, Amati C, and Vohra S. (2008). A review of workplace interventions that promote mental wellbeing in the workplace. : Institute of Occupational Medicine, pp.1-232. . | Narrative | Mental health and substance use |
| Groeneveld IF, Proper KI, van der Beek AJ, Hildebrandt VH, and van Mechelen W. (2010). Lifestyle-focused interventions at the workplace to reduce the risk of cardiovascular disease--a systematic review. Scandinavian journal of work, and environment & health, 36(3), pp.202-15. | Narrative | CVD prevention |
| Gudzune K, Hutfless S, Maruthur N, Wilson R, and Segal J. (2013). Strategies to prevent weight gain in workplace and college settings: a systematic review. Preventive medicine, 57(4), pp.268-77. | Narrative | Physical activity, nutrition |
| Harden A, Peersman G, Oliver S, Mauthner M, and Oakley A. (1999). A systematic review of the effectiveness of health promotion interventions in the workplace. Occupational medicine (Oxford, and England), 49(8), pp.540-8. | Narrative | Diverse |
| Hosking J, Macmillan A, Connor J, Bullen C, and Ameratunga S. (2010). Organisational travel plans for improving health. Cochrane Database of Systematic Reviews, (Issue 3), pp. | Narrative | Policy and scheduling |
| Hutchinson AD, and Wilson C. (2012). Improving nutrition and physical activity in the workplace: a meta-analysis of intervention studies. Health promotion international, 27(2), pp.238-49. | Meta-analysis | Physical activity, nutrition |
| Joyce K, Pabayo R, Critchley JA, and Bambra C. (2010). Flexible working conditions and their effects on employee health and wellbeing. Cochrane Database of Systematic Reviews: Reviews, Issue 2, pp. | Narrative | Policy and scheduling |
| Kahn-Marshall JL, and Gallant MP. (2012). Making healthy behaviors the easy choice for employees: a review of the literature on environmental and policy changes in worksite health promotion. Health Education and Behavior, 39(6), pp.752-776. | Narrative | Policy and scheduling |
| Kaspin LC, Gorman KM, and Miller RM. (2013). PRM51 Comprehensive Review of Employer-Sponsored Wellness Strategies and Their Economic and Health-Related Outcomes: Evidence Quality and Opportunities for Future Research. Population health management, 16(1), pp.14-21. | Narrative | Diverse |
| Knowlden AP, Ickes MJ, and Sharma M. (2014). Systematic analysis of tobacco treatment interventions implemented in worksite settings. Journal of Substance Use, 19(4), pp.283-294. | Narrative | Smoking cessation |
| Kremers S, Reubsaet A, and Martens M et al. (2010). Systematic prevention of overweight and obesity in adults: a qualitative and quantitative literature analysis. Obesity Reviews, 11(5), pp.371-379. | Meta-analysis | Physical activity, nutrition |
| Kuoppala J, Lamminpaa A, and Husman P. (2008). Work health promotion, job well-being, and sickness absences--a systematic review and meta-analysis. Journal of occupational and environmental medicine / American College of Occupational and Environmental Medicine, 50(11), pp.1216-27. | Narrative and meta-analysis | Diverse |
| Leeks KD, Hopkins DP, Soler RE, Aten A, and Chattopadhyay SK. (2010). Worksite-based incentives and competitions to reduce tobacco use. A systematic review. American journal of preventive medicine, 38(2 Suppl), pp.S263-74. | Narrative | Smoking cessation |
| MacEwen BT, MacDonald DJ, and Burr JF. (2015). A systematic review of standing and treadmill desks in the workplace. Preventive Medicine, 70C, pp.50-58. | Narrative | Physical activity |
| Maes L, Van Cauwenberghe E, Van Lippevelde W, Spittaels H, De Pauw E, Oppert JM, Van Lenthe FJ, Brug J, and De Bourdeaudhuij I. (2012). Effectiveness of workplace interventions in Europe promoting healthy eating: a systematic review. European journal of public health, 22(5), pp.677-83. | Narrative | Nutrition |
| Maher CG. (2000). A systematic review of workplace interventions to prevent low back pain. The Australian journal of physiotherapy, 46(4), pp.259-269. | Narrative | Musculoskeletal symptoms |
| Malik SH, Blake H, and Suggs LS. (2014). A systematic review of workplace health promotion interventions for increasing physical activity. British journal of health psychology, 19(1), pp.149-80. | Narrative | Physical activity |
| Martin A, Sanderson K, and Cocker F. (2009). Meta-analysis of the effects of health promotion intervention in the workplace on depression and anxiety symptoms. Scandinavian Journal of Work, and Environment and Health, 35(1), pp.7-18. | Meta-analysis | Mental health and substance use |
| Matson-Koffman DM, Brownstein JN, Neiner JA, and Greaney ML. (2005). A site-specific literature review of policy and environmental interventions that promote physical activity and nutrition for cardiovascular health: what works?. American Journal of Health Promotion, 19(3), pp.167-193. | Narrative | Physical activity, nutrition, CVD prevention |
| McLeod J. (2010). The effectiveness of workplace counselling: A systematic review. Counselling & Psychotherapy Research, 10(4), pp. | Narrative | Mental health and substance use |
| Mehta S, Dimsdale J, Nagle B, Holub CK, Woods C, Barquera S, and Elder JP. (2013). Worksite interventions: improving lifestyle habits among Latin American adults. American Journal of Preventive Medicine, 44(5), pp.538-542. | Narrative | Physical activity, nutrition |
| Micucci S, and Thomas H. (2007). The Effectiveness of Multi-faceted Health Promotion Interventions in the Workplace to Reduce Chronic Disease. : , pp. Available at: http://old.hamilton.ca/phcs/ephpp/Research/Full-Reviews/2007/InterventionsForWorkplaceReductionOfChronicDiseases.pdf. | Narrative | Comprehensive |
| Milner A, Page K, Spencer-Thomas S, and Lamotagne AD. (2014). Workplace suicide prevention: a systematic review of published and unpublished activities. Health promotion international, , pp. | Narrative | Mental health and substance use |
| Moher M, Hey K, and Lancaster T. (2005). Workplace interventions for smoking cessation. The Cochrane database of systematic reviews, (2), pp.CD003440. | Narrative | Smoking cessation |
| Montano D, Hoven H, and Siegrist J. (2014). A meta-analysis of health effects of randomized controlled worksite interventions: does social stratification matter?. Scandinavian journal of work, and environment & health, 40(3), pp.230-4. | Meta-analysis | Diverse |
| Montano D, Hoven H, and Siegrist J. (2014). Effects of organisational-level interventions at work on employees' health: a systematic review. BMC public health, 14, pp.135. | Narrative | Policy and scheduling |
| Mozaffarian D, Afshin A, Benowitz NL, Bittner V, Daniels SR, Franch HA, Jacobs DR Jr, Kraus WE, Kris-Etherton PM, Krummel DA, Popkin BM, Whitsel LP, and Zakai NA. (2012). Population approaches to improve diet, physical activity, and smoking habits: a scientific statement from the American Heart Association. Circulation, 126(12), pp.1514-63. | Narrative | Physical activity, nutrition, smoking cessation |
| Ni Mhurchu C, Aston LM, and Jebb SA. (2010). Effects of worksite health promotion interventions on employee diets: a systematic review. BMC public health, 10, pp.62. | Narrative | Nutrition |
| NICE (2008). Workplace interventions that are effective for promoting mental wellbeing. Synopsis of the evidence of effectiveness and cost-effectiveness. | Narrative | Mental health and substance use |
| Odeen M, Magnussen LH, Maeland S, Larun L, Eriksen HR, and Tveito TH. (2013). Systematic review of active workplace interventions to reduce sickness absence. Occupational medicine (Oxford, and England), 63(1), pp.7-16. | Narrative | Diverse |
| Ogilvie D, Foster C E, Rothnie H, Cavill N, Hamilton V, Fitzsimons C F, and Mutrie N. (2007). Interventions to promote walking: systematic review. BMJ, 334(7605), pp.1204. | Narrative | Physical activity |
| Ojo O, Verbeek J, Rasanen H, Heikkinen J, Isotalo L, Mngoma N, and Ruotsalainen E. (2011). Interventions to reduce risky sexual behaviour for preventing HIV infection in workers in occupational settings. Cochrane Database of Systematic Reviews, (Issue 12), pp. | Meta-analysis | Sexual health |
| Osilla KC, Van Busum K, Schnyer C, Larkin JW, Eibner C, and Mattke S. (2012). Systematic review of the impact of worksite wellness programs. The American journal of managed care, 18(2), pp.e68-81. | Narrative | Diverse |
| Parks KM, and Steelman LA. (2008). Organizational wellness programs: A meta-analysis. Journal of Occupational Health Psychology, 13(1), pp.58-68. | Meta-analysis | Physical activity, comprehensive |
| Peersman G, Harden A, and Oliver S. (1998). Effectiveness of health promotion interventions in the workplace: a review. Health Promotion Effectiveness Reviews, , pp.93. | Narrative | Diverse |
| Pelletier KR. (1997). Clinical and cost outcomes of multifactorial, cardiovascular risk management interventions in worksites: a comprehensive review and analysis. J Occup Environ Med, 39(12), pp.1154-69. | Narrative | CVD prevention |
| Pelletier KR. (2005). A review and analysis of the clinical and cost-effectiveness studies of comprehensive health promotion and disease management programs at the worksite: update VI 2000-2004. J Occup Environ Med, 47(10), pp.1051-8. | Narrative | Comprehensive |
| Pelletier KR. (2011). A review and analysis of the clinical and cost-effectiveness studies of comprehensive health promotion and disease management programs at the worksite: update VIII 2008 to 2010. J Occup Environ Med, 53(11), pp.1310-31. | Narrative | Comprehensive |
| Proper K I, Koning M, van der Beek A J, Hildebr , t V H, Bosscher R J, and van Mechelen W. (2003). The effectiveness of worksite physical activity programs on physical activity, physical fitness, and health. Clinical Journal of Sports Medicine, 13(2), pp.106-17. | Narrative | Physical activity |
| Richardson KM, and Rothstein HR. (2008). Effects of occupational stress management intervention programs: A meta-analysis. Journal of Occupational Health Psychology, 13(1), pp. | Meta-analysis | Mental health and substance use |
| Rongen A, Robroek SJ, van Lenthe FJ, and Burdorf A. (2013). Workplace health promotion: a meta-analysis of effectiveness. American journal of preventive medicine, 44(4), pp.406-15. | Meta-analysis | Diverse |
| Schröer S, Haupt J, and Pieper C. (2014). Evidence-based lifestyle interventions in the workplace--an overview. Occupational Medicine, 64(1), pp. | Narrative | Diverse |
| Segui-Gomez M. (2000). Evaluating worksite-based interventions that promote safety belt use. Am J Prev Med, 18(4 Suppl), pp.11-22. | Narrative | Diverse |
| Smedslund G, Fisher KJ, Boles SM, and Lichtenstein E. (2004). The effectiveness of workplace smoking cessation programmes: a meta-analysis of recent studies. Tobacco control, 13(2), pp.197-204. | Meta-analysis | Smoking cessation |
| Steyn NP, Parker W, Lambert EV, and Mchiza Z. (2009). Nutrition interventions in the workplace: evidence of best practice. South African Journal of Clinical Nutrition, 22(3), pp.111-117XPT: Journal article. | Narrative | Nutrition |
| Tan L, Wang MJ, Modini M, Joyce S, Mykletun A, Christensen H, and Harvey SB. (2014). Preventing the development of depression at work: a systematic review and meta-analysis of universal interventions in the workplace. BMC medicine, 12, pp.74. | Meta-analysis | Mental health and substance use |
| Thomson CA, and Ravia J. (2011). A systematic review of behavioral interventions to promote intake of fruit and vegetables. Journal of the American Dietetic Association, 111, pp.1523-1535. | Narrative | Nutrition |
| To QG, Chen TT, Magnussen CG, and To KG. (2013). Workplace physical activity interventions: a systematic review. American journal of health promotion : AJHP, 27(6), pp.e113-23. | Narrative | Physical activity |
| Torbeyns T, Bailey S, Bos I, and Meeusen R. (2014). Active workstations to fight sedentary behaviour. Sports Med., 44(9), pp.1261-73. | Narrative | Physical activity |
| Tveito T H, Hysing M, and Eriksen H R. (2004). Low back pain interventions at the workplace: a systematic literature review. Occup.Med.(Lond), 54(1), pp.3-13. | Narrative | Musculoskeletal symptoms |
| van Poppel MN, Hooftman WE, and Koes BW. (2004). An update of a systematic review of controlled clinical trials on the primary prevention of back pain at the workplace. Occupational medicine (Oxford, and England), 54(5), pp.345-52. | Narrative | Musculoskeletal symptoms |
| Verweij LM, Coffeng J, van Mechelen W, and Proper KI. (2011). Meta-analyses of workplace physical activity and dietary behaviour interventions on weight outcomes. Obesity reviews : an official journal of the International Association for the Study of Obesity, 12(6), pp.406-29LID. | Meta-analysis | Physical activity, nutrition |
| Vuillemin A, Rostami C, Maes L, Van Cauwenberghe E, Van Lenthe FJ, Brug J, De Bourdeaudhuij I, and Oppert JM. (2011). Worksite physical activity interventions and obesity: a review of European studies (the HOPE project). Obesity Facts, 4(6), pp.479-488. | Narrative | Physical activity |
| Webb G, Shakeshaft A, Sanson-Fisher R, and Havard A. (2009). A systematic review of work-place interventions for alcohol-related problems. Addiction (Abingdon, and England), 104(3), pp.365-77. | Narrative | Mental health and substance use |
| World Health Organization. (2009). Interventions on Diet and Physical Activity: What Works. | Narrative | Physical activity, nutrition |
